# Histone *H3* genotyping refines clinico-radiological diagnostic and prognostic criteria in DIPG

**DOI:** 10.1007/s00401-016-1568-7

**Published:** 2016-04-01

**Authors:** David Castel, Jacques Grill, Marie-Anne Debily

**Affiliations:** UMR8203 “Vectorologie and Thérapeutiques Anticancéreuses”, CNRS, Gustave Roussy, Univ. Paris-Sud, Université Paris-Saclay, 94805 Villejuif, France; Département de Cancérologie de l’Enfant et de l’Adolescent, Gustave Roussy, Univ. Paris-Sud, Université Paris-Saclay, 94805 Villejuif, France; Université d’Evry-Val d’Essonne, Boulevard François Mitterrand, Evry, 91025 France

We thank our colleagues for their comments on our recent publication [1], especially on the use of histone H3 K27M mutations as prognostic biomarker in addition to its use for diagnostic purposes [2]. We would like, nonetheless, to address some of the points raised in their letter.

To test how this biomarker performed in our cohort, we compared it to the “DIPG Risk Score” previously published by Jansen et al. [4]. We acknowledge that we did not analyse the same population of patients, as our cohort was composed of systematically biopsied patients fulfilling all criteria for a DIPG. In particular, we applied a strict definition of DIPG based on a typical radiology and a short symptom duration (<6 months) as widely accepted criteria for inclusion in trials [1, 3, 5]. We had mistakenly indicated symptoms duration below 3 months in our manuscript, and we do apologize for the confusion introduced. Of note, only 5 cases in the whole cohort (*n* = 91) had symptoms duration between 3 and 6 months. Patients with long symptoms duration (i.e. over 6 months) were never included in the cohort—none were therefore ‘excluded from the analysis’. These cases with either prolonged duration or atypical features on MRI deserve a specific attention to define which are true DIPG and which are not, with the help of a proper biologic identification (detection of K27M mutation, H3K27 trimethylation loss…). A short symptoms duration is always considered as one of the hallmarks of DIPG, but it may be difficult in some cases to define which is the first symptom of the disease, especially when this symptom has not a clear neurologic location (e.g. behavioural changes). It remains to be confirmed how frequent histone H3-K27M mutations are in patients with prolonged symptoms histories in comparison with patients with shorter histories as in our cohort.

In all, we transposed the “DIPG risk score” calculation to our cohort thus representing a restricted subset of infiltrative brainstem tumours compared to the one of Jansen et al., but with histologically and biologically confirmed DIPG. In the survival analysis using the “DIPG Risk score” presented in Figure S7I, we used all DIPG cases with complete demographical, survival and MRI contrast enhancement data (*n* = 81, and not 60 as quoted in the letter; see Figure S1), independently of their histone mutation status, hence including wild-type and H3.2-K27M tumours. The 10 cases that were excluded from the “DIPG Risk Score” evaluation had missing clinical data impeding its calculation.

As for the calculation of the impact of the H3.1- or H3.3-K27M mutation on survival, we logically excluded the 12 cases not showing these genotypes. Of note, survival curve of our DIPG patients where a histone H3 mutation was not identified was as bad as those of the DIPG patients with histone H3.1 and H3.3-K27M mutations (see figure S7f).

As underlined by Jansen et al, we indeed had very few patients in the “standard risk group” because our inclusion criteria were strictly applied (as discussed earlier), and we did not have patients with long symptoms duration—which may not correspond to the strict definition of DIPG that we have used, and neither infant.

In the multivariate analysis that we could apply on cases where both the “DIPG risk score” and the “histone H3-K27 M status” could be defined (*n* = 69), the histone status was the strongest predictor for survival as stated in the body of the text (unfortunately in the “Materials and methods” section). The clinico-radiological “DIPG Risk Score” did not completely fall out of the multivariate model albeit its contribution was only marginal. At least three elements that define the “DIPG Risk Score” could not have a contribution in the calculation in our cohort. First, the symptoms duration was always below 6 months in our case (and below 3 months for all but 5 cases in the whole cohort). Second, the chemotherapy treatment had no effect on survival in our dataset (see Figure S7H). Lastly, DIPG cases corresponding to our strict definition are rarely found in children below the age of three.

We acknowledge that we did not compare the performance of the “DIPG risk score” to the histone mutation stratification on the validation cohort from Wu et al. [6]. We used publically available DIPG data, and unfortunately few studies included detailed information related to age, survival and radiological data together with histone mutation status, that would have allowed the calculation of the “DIPG risk score” in parallel. Our cohort is so far the only public dataset allowing the comparison of histone H3 mutational status vs. the “DIPG risk score”, as all other published ones have missing data either on the biology or radiology.

Finally, what we proposed in our original publication goes beyond the strict descriptive stratification of patients at diagnosis, by unravelling a molecular origin in DIPG subgrouping based on Histone H3.1 and H3.3 K27 M mutations (yet excluding *de facto* wild-type DIPG). It has been clearly demonstrated in the paediatric brain tumour field that molecular analysis of tumours allows for a more accurate diagnosis both based on genetic (mutational or transcriptional) or epigenetic (i.e. DNA methylation) data. As stated by Jansen et al., all patients do not benefit from such analyses; we think, however, that probing the histone mutation status at diagnosis is the most unequivocal diagnostic and prognostic criteria for DIPG. This is in concordance with the evolution of the 2016 WHO classification, which includes a specific entity defined as “diffuse midline glioma, H3-K27M mutant”, and thus a biological definition of the disease rather than solely based on the clinical and radiological presentation.

It is not our intention to consider that clinical and radiological criteria do not improve prognostic models based on the biology only, since clinical and radiological characteristics correspond to the phenotypic correlates of the biology. Our work was meant to emphasize the usefulness of the biopsy at diagnosis to provide diagnostic and prognostic information on DIPG origin and outcome.
